# Distal femoral varus osteotomy: results of the lateral open-wedge technique without bone grafting

**DOI:** 10.1007/s00264-018-4216-0

**Published:** 2018-11-13

**Authors:** Alexander Kolb, Verena Isak, Gerhard M. Hobusch, Catharina Chiari, Reinhard Windhager

**Affiliations:** Department of Orthopedics and Trauma Surgery, Medical University of Vienna, Vienna, Austria

**Keywords:** Genu valgum, Femoral osteotomie, Open wedge

## Abstract

**Background:**

The lateral opening wedge distal femoral osteotomy (LOWDFO) to reconstruct knee alignment in patients with genu valgum originating in the distal femur has gained importance within the last years.

**Purpose:**

To analyze clinical and radiographic outcome of patients treated with LOWDFO with respect to bone healing without grafting and patient age.

**Material and methods:**

Twenty-two consecutive patients with genu valgum corrected with 23 LOWDFOs using a Tomofix-locking plate were retrospectively analyzed (mean age 23.7 years). Clinical evaluation was based on pre- and post-operative KOOS scores. A pre- and post-operative radiographic assessment, including MAD, mLDFA, LLD, bone healing, and patella parameters, was performed. Differences between subgroups (age, bone grafting) were analyzed.

**Results:**

The restoration of MAD and mLDFA resulted in significantly improved post-operative KOOS_5_ scores in younger and older patients (*p* = 0.001). Bone healing without bone grafting was reliable in all patients. The leg length was significantly increased post-operatively (*p* = 0.001). The Blackburne-Peel ratio was significantly reduced to more normal values post-operatively (*p* < 0.001).

**Conclusion:**

LOWDFO without bone grafting is a reliable procedure representing a promising treatment option particularly in young patients with genu valgum. Besides correction of the MAD, a significant leg length increase and additional patella stability can be expected.

## Introduction

Osteotomies around the knee are an accepted option for the treatment of varus and valgus malalignment of the lower extremity. When malalignment is associated with unicompartmental damage of the knee, the mechanical axis is shifted to transfer weight load from the damaged to the healthy compartment. In the absence of symptoms, knee osteotomies are also an option to restore a physiologic mechanical axis in order to prevent ongoing joint damages. Depending on the origin of the deformity, which needs to be analyzed by the malalignment test according to Paley [1], the corrective osteotomy is performed on the proximal tibia or distal femur as a closing or opening wedge osteotomy. The majority of cases of valgus malalignment show a reduced mLDFA, which is responsible for the malalignment. This type of deformity can be addressed by an opening wedge osteotomy of the distal femur [2].

Interestingly, the open-wedge technique is broadly used for the tibial correction of varus deformities [3], while the femoral open-wedge technique was discussed as being controversial. Basically, reports on specific implants used for the lateral opening wedge distal femoral osteotomy like the Tomofix plate (Synthes GmbH, Oberdorf, Switzerland) are rare, relied on different techniques of bone grafting, and have reported controversial results [4–7].

This study aimed to (1) analyze the results of the lateral opening wedge distal femoral osteotomy without bone grafting, (2) to assess differences between pre- and post-operative knee function and mechanical alignment including effects on leg length and patellar parameters, (3) to assess the influence of patient age, and (4) to assess complications related to bone healing, local pain, and surgical site infection.

## Material and methods

A retrospective, cohort study was performed on all patients, who underwent lateral opening wedge distal femoral osteotomy at our institution between 2006 and 2016. The inclusion criteria were LOWDFO performed at our institution because of genu valgum associated with knee pain or limited knee function due to valgus deformity. The pre-operative valgus deformity had to meet the following criteria: MAD below − 10 mm (normal value 9.7 ± 6.8 mm [1]) combined with a reduced mLDFA (normal value 88°). Patients with any known neurological or metabolic disorder potentially affecting knee function or bone healing were excluded. Twenty-two patients (18 female; 4 male) were identified from clinical records and were included in this study. One female patient was treated bilaterally giving a total of 23 included open-wedge osteotomies of the distal femur. The patients were informed about the possibility of participating in this study. Detailed information about the study aims and procedures was provided to the patients prior to recruitment, and participation was with written informed consent. One female patient, who was unavailable for follow-up on multiple callings, was excluded from the study. Participants’ ages at the time of surgery ranged from 12.8 to 48.6 years (mean age 23.7 years). The mean follow-up was 65 months (range 13 to 125 months). Using a cut-off of 20 years, two age groups were defined (younger patients (aged 13–20 years) and older patients (aged 21–49 years)) to analyze differences of treatment effects.

Knee function was assessed preoperatively and at the time of follow-up by the Knee injury and Osteoarthritis Outcome Score (KOOS), which is a self-reporting questionnaire consisting of 5 subscales: pain, other symptoms, activity in daily living (ADL), function in sport and recreation, and knee-related quality of life (QOL) [8]. For each subscale, a score between 100 (no symptoms) and 0 (extreme symptoms) is calculated. The KOOS_5_, a composite score calculated from the KOOS as mean of the five subscales, was used to measure the primary outcome [9]. The five KOOS subscales are used as secondary outcome measures to support a clinically relevant interpretation of the results.

A time of follow-up a standardized clinical examination was performed including the assessment of local pain in the lateral distal femur measured by numeric rating scale (NRS) [10].

## Pre-operative planning

In patients without radiographic signs of osteoarthritis, a reconstruction of the neutral mechanical axis was planned, whereas in cases with degenerative changes in the lateral compartment, an overcorrection of the mechanical axial to the medial aspect of the joint was planned.

Preoperative planning was done based on bipedal anteroposterior (AP) long-leg radiography [1]. After the malalignment test, the physiological distal and proximal mechanical axes of the femur were constructed, to determine the centre of rotation and angulation (CORA) at the intersection of these lines [1]. Based on this analysis, the osteotomy including the width of the desired opening wedge was planned. Patients showing a position of the CORA which was inappropriate for DFO requiring additional correction or procedures were not included in the study. A translation of the distal fragment due to an acceptable discrepancy of the CORA and the osteotomy site was not intended [11].

## Operative technique and post-operative treatment

All patients were placed in supine position. A longitudinal incision was made over the lateral distal femur using a subvastus approach to the femur. The Tomofix plate was placed epiperiostally, and the position of the plate in relation to the distal femur was checked in two planes using fluoroscopic control. This position was marked temporarily with a K-wire placed in the distal central screw hole of the plate, and a K-wire was placed under fluoroscopic control slightly convergent to the joint line in the direction of the planned osteotomy, before removing the plate. For rotational control, two small drill holes were made proximal and distal adjacent to the level of the planned osteotomy. An oscillating saw was used for the osteotomy in the direction of the K-wire, under protection of the posterior and anterior soft tissues with flexible aluminum retractors. Care was taken to avoid a complete transection of the medial cortex. The lateral osteotomy was slowly opened up to the planned distance, and a spacer was used to temporarily fix the position. The Tomofix plate was now fixed distally and proximally using three bi-cortical and one mono-cortical screw in the proximal segment. In five selected cases, ChronOS® (Synthes, Inc) bone graft substitute was used to fill the osteotomy gap.

All patients were mobilized post-operatively according to a standardized protocol prescribing partial weight-bearing for six weeks and continuous passive motion (CPM). Weight-bearing was gradually increased achieving full weight-bearing usually after ten weeks.

### Radiological analysis

**Fig. 1:**
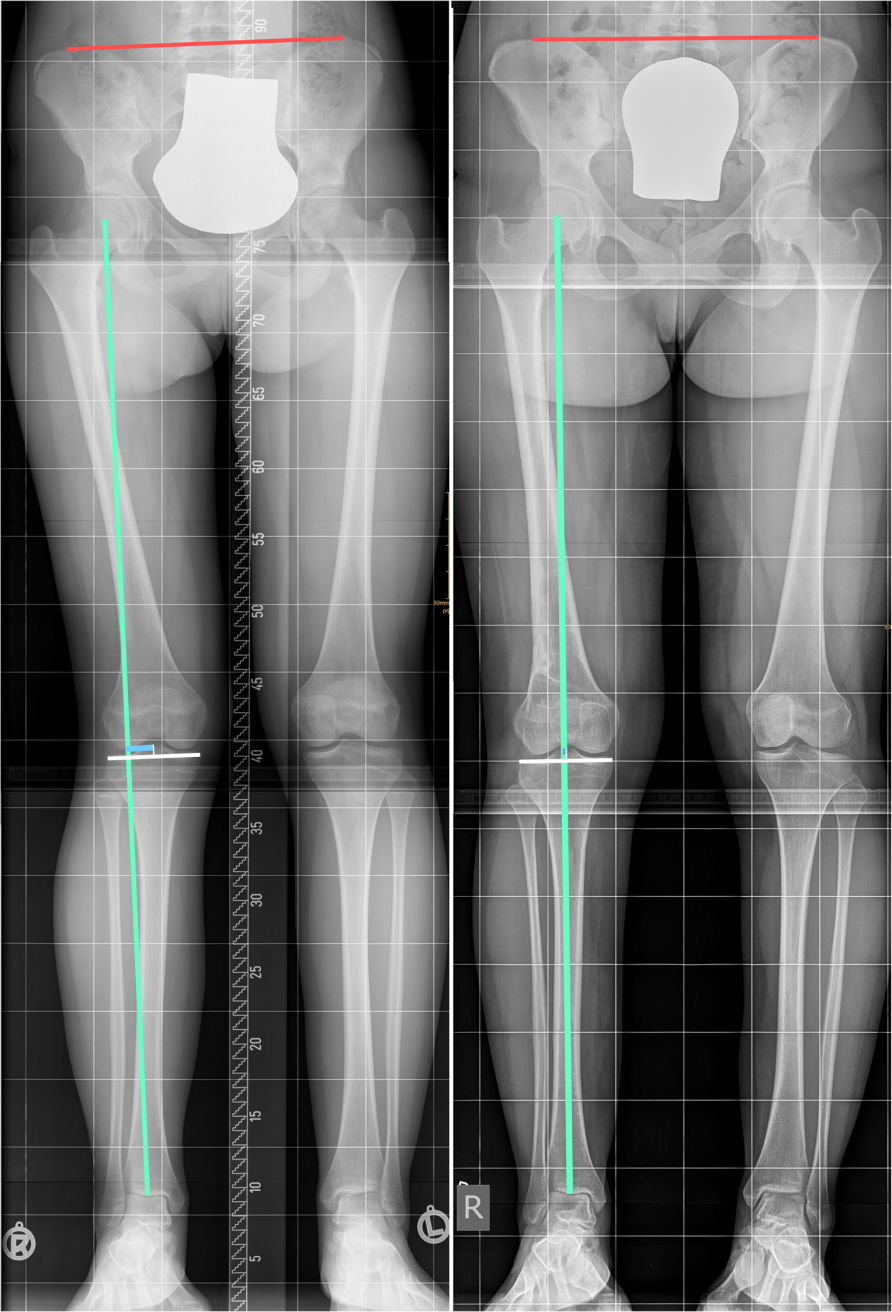
Comparison of pre- and post-operative long leg standing radiographs: mechanical axis of the leg (green line), tibial plateau width (white line with mark at the midpoint), MAD (blue line), and LLD (red line) radiographs

**Fig. 2:**
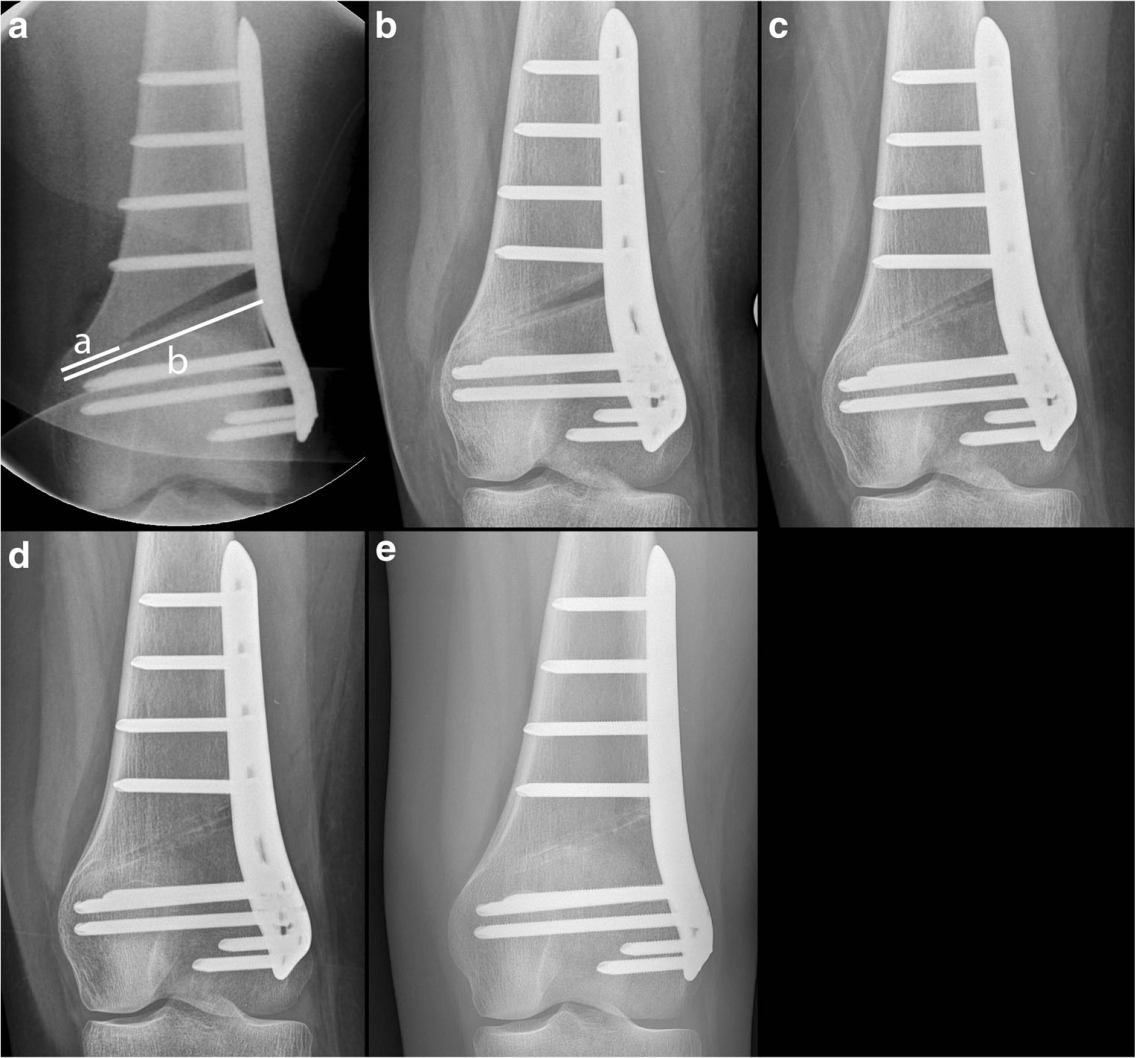
Assessment of bone healing according to Staubli and Schröter [12, 13]. **a** Direct postoperatively, after 6 weeks (**b**), 3 month (**c**), after 6 month (**d**), and 1 year post-operatively (**e**). The width of the opened wedge showing bone healing (distance a) and width of the osteotomy from lateral to medial (distance b) were measured. The percentage of the osteotomy gap showing bone healing was calculated (distance a/distance b * 100)

**Fig. 3:**
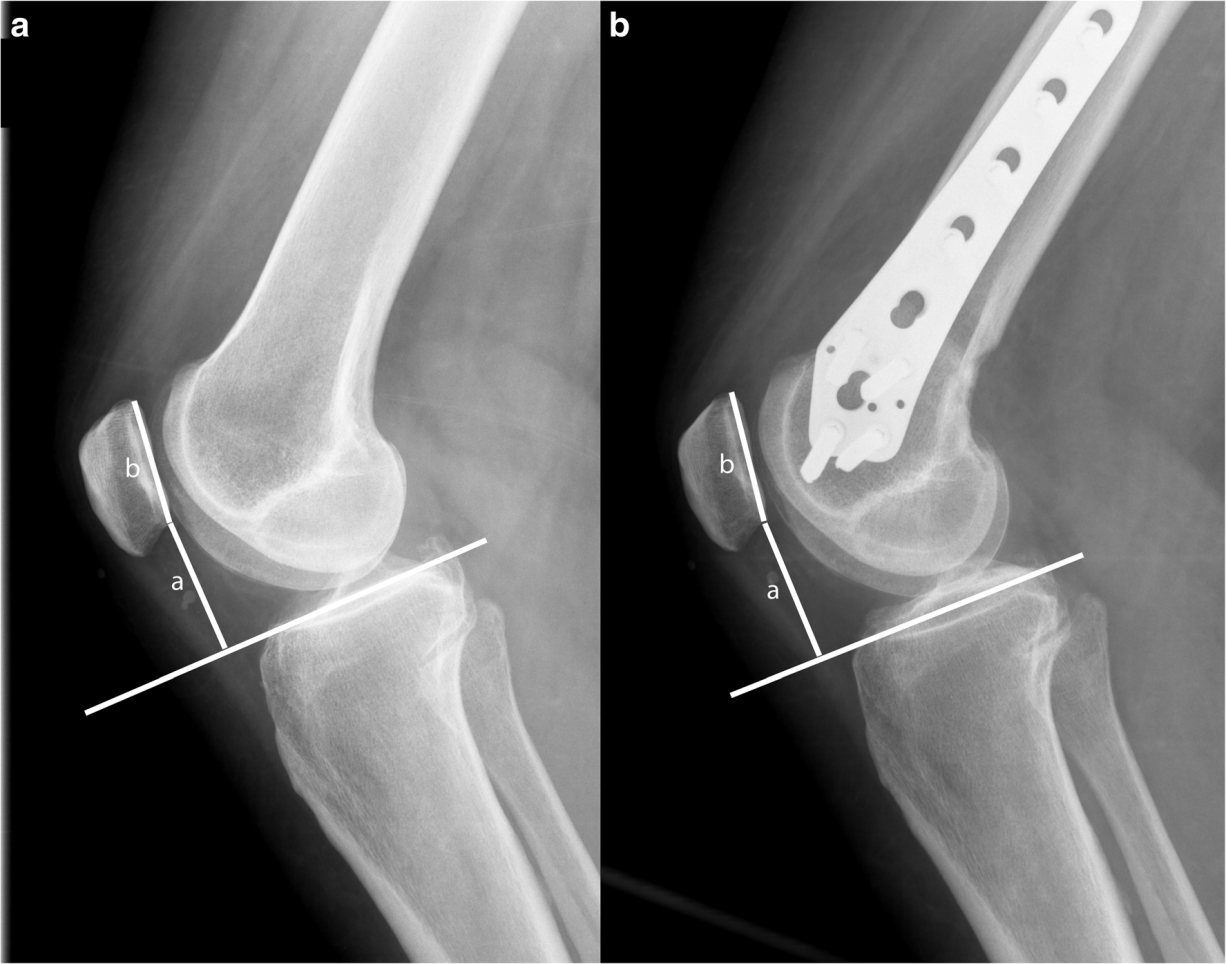
Measurement of the patellar height on lateral radiographs pre-operatively (**a**) and post-operatively (**b**); measurement of the perpendicular height of the lower end of the articular surface of the patella from the tibial plateau line (a) and length of the articular surface of the patella (b). The Blackburne-Peel ratio is defined as a/b [15]

In all patients, bipedal anteroposterior (AP) long-leg radiography with the patellae facing forward and the knee in full extension, and standard AP and lateral radiographs of the knee in 30° flexion were obtained pre- and post-operatively. The mechanical axis deviation (MAD), mechanical lateral distal femoral angle (mLDFA), and leg length discrepancy (LLD) were measured on the AP long-leg radiography [1] (see Fig. 1). Bone healing was assessed radiographically using a modification of the method suggested by Staubli and Schröter et al. for the measurement in patients with open-wedge high tibial osteotomy [12, 13] (see Fig. 2). The radiographic evaluation was also focused on the patellofemoral compartment. Therefore, the Blackburne-Peel ratio was measured pre- and post-operatively. The Blackburne-Peel ratio was used to assess the patellar height on the lateral radiographs (normal values between 0.54 and 1.06) [14] (see Fig. 3).

### Statistical analysis

Data were processed using the SPSS 25 software (SPSS Inc., Chicago, IL, USA). Differences between pre- and post-operative measurements were assessed using the paired *t* test and Wilcoxon matched-pairs signed-rank test where appropriate. Differences between groups were compared using the independent *t* test or the Mann-Whitney *U* test as appropriate. All datasets were assessed for normal distribution using the Kolmogorov-Smirnov test and evaluation of quantile-quantile-plots. The level of significance was defined as *p* < 0.05.

## Results

Mean KOOS_5_ changed from 60.0 (range 13.8 to 100.0) pre-operatively to 81.9 (range 49.4 to 100.0) post-operatively. This improvement was statistically significant (*p* = 0.001). All five KOOS subscales improved significantly (*p* = 0.001 to 0.013, see Table 1). The comparison of the two age groups showed no significant differences in the improvement of KOOS_5_ or KOOS subscales (*p* = 0.140 to *p* = 0.790), whereas higher pre- and post-operative mean KOOS_5_ scores were found in younger patients (statistically significant, *p* = 0.052 and *p* = 0.029, see Table 2).

**Table 1:** Summary of results of LOWDFO); significant values are marked with asterisk

	Pre-operative mean (SD)	Post-operative mean (SD)	Mean difference	*p* value
KOOS 5	60.0 (27.2)	81.9 (16.2)	− 21.9	0.001*
KOOS pain	62.7 (26.8)	84.2 (14.3)	− 21.5	0.002*
KOOS sym	67.7 (24.2)	83.5 (17.3)	− 15.8	0.003*
KOOS ADL	69.3 (29.6)	89.1 (13.2)	− 19.8	0.013*
KOOS sport	47.6 (34.4)	73.9 (27.5)	− 26.3	0.001*
KOOS QOL	52.6 (35.3)	78.6 (23.0)	− 26.0	0.002*

**Table 2:** Comparison of age groups: (A) younger patients (age < =20 years) and (B) older patients (age > 20 years); significant values are marked with asterisk

	Age group A (*N* = 13) mean (SD)	Age group B (*N* = 5) mean (SD)	Mean difference	*p* value
KOOS 5 pre-operative	68.9 (24.8)	41.2 (25.8)	− 27.7	0.052*
KOOS 5 post-operative	88.1 (9.6)	71.1 (21.1)	− 17.0	0.029*
KOOS 5 improvement	19.2 (23.7)	30.0 (24.4)	10.8	0.402
KOOS pain improvement	18.8 (25.2)	31.1 (27.9)	12.3	0.428
KOOS sym improvement	11.5 (19.5)	30.0 (23.4)	18.5	0.140
KOOS ADL improvement	15.4 (25.1)	37.1 (36.7)	21.7	0.254
KOOS sport improvement	25.8 (31.0)	23.0 (29.9)	− 2.8	0.727
KOOS QOL improvement	24.5 (31.3)	28.8 (24.4)	4.2	0.790

Mean knee alignment measured by MAD changed from − 34.8 mm valgus (range − 70.0 to − 11.0 mm) pre-operatively to − 1.9 (range − 32.1 to 12.5 mm). This difference was statistically significant (*p* < 0.001). Mean mechanical lateral distal femoral angle (mLDFA) changed from 81.1° valgus (range 74.5 to 86.3°) pre-operatively to 88.5° (range 82.5° to 92.7°). This difference was statistically significant (*p* = 0.023). The mean leg length discrepancy (LLD) changed from − 6.4 mm (range − 25.0 to 9.0 mm) pre-operatively to 1.5 (range − 11.0 to 16.0 mm) post-operatively. This difference was statistically significant (*p* = 0.001).

The patella height assessed by the Blackburne-Peel ratio was statistically significantly reduced from pre-operative (mean 1.01, SD 0.18) to post-operative measurements (mean 0.90, SD 0.18, *p* < 0.001).

The mean osteotomy gap height measured post-operatively was 9.0 mm (range 4.6 to 15.5 mm). In all patients, a progressive bone healing of the osteotomy gap was observed. Certainly, there was no new bone formation in the osteotomy gap post-operatively. However, on the anteroposterior radiographs, the complete gap was not visible, resulting in a mean gap filling ratio of 25.6% (range SD 11.2%). The mean osteotomy gap filling rates of patients without bone grafting increased with time to 40.2% (SD 19.1%) at six weeks, 54.2% (SD 26.0%) at 12 weeks, 72.3% (SD 27.2%) at six months, 93.2% (SD 16.1%) at 12 months, and 98.0% (SD 8.1%) at 18 months. The comparison to the five cases with ChronOS® bone graft substitute gap filling revealed no statistical significant difference (*p* = 0.225 to 0.925). The height of the osteotomy gap between these groups did not differ statistically significantly (*p* = 0.157).

When bone healing was judged to be complete, patients were scheduled for plate removal, which was done at the earliest 14 months post-operatively (mean23 month, range 14 to 35 months).

Mild local pain (NRS 1 to 2) in the lateral distal femur was registered in four of 20 cases (20%), while in two cases, the question was not answered. However, the local pain diminished in all cases after plate removal. None of the patients had to be scheduled for revision surgery due to local pain or other complications. Cases of surgical site infection or thrombosis were not observed in the cohort.

## Discussion

Our retrospective cohort study aimed to analyze the clinical and radiological results of patients treated by lateral opening wedge osteotomy without bone grafting. The central findings were that (1) good clinical results based on a reliable bone healing without bone grafting can be achieved, (2) knee function measured by KOOS and mechanical parameters were improved significantly, (3) promising results in young patients can be achieved, and (4) complications were uncommon.

The clinical outcome evaluated by KOOS_5_ and the five KOOS subscales (pain, other symptoms, function in daily living, function in sport and recreation, and knee-related quality of life) was significantly improved. When comparing the two age groups of younger patients (age ≤ 20 years) and older patients (age > 20), we did not find significant differences of treatment effects measured by KOOS_5_. This is an interesting finding, as one would expect to have a lower mid-term benefit in younger patients, due to higher pre-operative scores. Therefore, in the context of statistically significant higher pre- and post-operative scores of younger patients, the tendency to lower treatment effects in this group represents no limitation for LOWDFO in young patients. However, the analysis of the five KOOS subscales provides additional insights: First, no significant differences between the age groups were found for any of the five subscales. However, the trend to smaller treatment effects is seen in all but one subscale: Only the KOOS sport subscale shows similar treatment effects in both groups suggesting that an especially notable benefit of LOWDFO in younger patients is an improved sporting ability.

In summary, the clinical results of our cohort are comparable to those studies, who reported reliable results on LOWDFO [5–7, 16]. As a difference to former studies, our cohort includes especially younger patients as discussed above (for example Elattar at al. (range 22 to 72 years) [7], Jacobi at al. (46 + −3.1 years) [4], Dewilde et al. (range 30 to 51 years) [16], and Saithna et al. (range 28 to 58 years) [6]). Cameron et al. [5] focused on two age groups comparing patients with arthritis (mean age 41 years, SD 9) and patients treated for joint preservation (mean age 26 years, SD 8). Similar to our results, a higher treatment effect in the arthritis group was reported in the group of elder patients, but in contrast to our results, this group also ended up with higher IKDC scores. This difference to our results might be related to additional procedures performed in the group treated for joint preservation (chondroplasty, osteochondral allografting, and meniscal allografting). Here, the younger age group of our cohort defined by a cut-off of 20 years was notably younger and did not require additional joint reconstruction procedures, suggesting that an early reconstruction of limb alignment is favourable. However, due to the limited evidence on medial closing-wedge distal femoral osteotomies especially in young patients under 20 years of age, a direct comparison of our results to this common procedure is not possible. While, recent reports on the comparison of open- and closed-wedge distal femoral varus osteotomy performed in patients with valgus arthritis of the knee have shown similar outcomes for both procedures [17, 18], further studies are needed to examine whether this finding is also true for young patients.

In contrast to our study, Jacobi at al. has reported unfavourable results [4] due to complications like local tissue irritation and delayed bone healing. Most reports on lateral opening wedge distal femoral osteotomy are based on a technique including bone grafting of the opening wedge osteotomy [4, 5, 7, 16]. Here, with the exception of Jacobi et al. who reported a non-union rate of 7.1%, a reliable low rate of non-unions (0 to 3%) was reported [5, 16]. Reports on LOWDFO without bone grafting are rare. Saithna et al. [6] used bone grafting only in cases of an opening wedge height above 12 mm, reporting a complication rate of 19% related to bone healing (delayed union, non-union, loss of correction). However, it has to be mentioned that Saithna et al. included patients treated with the Puddu plate (Arthrex Inc., Naples, FL) and the Tomofix plate, and that no specific number for the Tomofix plate was reported. Our cohort was also focused on patients treated by LOWDFO without bone grafting. However, in contrast to Saithna et al., we did not see complications related to bone healing. Moreover, the comparison of patients treated without bone grafting to the included five patients with bone grafting revealed no statistically significant difference in terms of bone healing assessed by mean osteotomy gap filling rates or osteotomy gap heights. Considering that medial opening-wedge high tibial osteotomies are commonly performed without bone grafting producing reliable results [3], which represents a technically similar procedure, our result of reliable bone healing is not surprising. Here, our findings suggest that the benefit of bone grafting is limited in lateral opening-wedge distal femoral osteotomies. Moreover, our findings are supported by Liska et al., who reported comparable results in a slightly older cohort using a similar technique [19].

Another criticism on LOWDFO is local pain due to soft tissue irritation caused by the metal plate, which was discussed controversially. Here, reports on lateral opening wedge distal femoral osteotomy use different definitions of soft tissue irritation or lack to provide detailed information. However, a rate of 22 to 86% can be found in the literature [4–6, 16]. Jacobi et al. reported local irritation of the iliotibial tract requiring implant removal in 86% of the patients with the Tomofix plate [4], while Dewilde et al. reported a rate of 22% using the Puddu plate, which was explained with the lower profile of the Puddu plate [16]. Comparing less invasive stabilization system plating to retrograde intramedullary nailing, Özcan et al. suggest that tissue irritation due to metal plates might compromise the clinical outcome measured by KOOS [20]. In our study, mild local pain (NRS 1 to 2) was observed in 20% of the patients while the plate was in situ. In contrast to other authors, plate removal was planned in all cases regardless of local symptoms. However, the local pain diminished in all cases after implant removal.

To date, the evidence on the influence of valgus alignment on patella tracking and stability is limited. McWalter et al. described the relationship of varus- or valgus alignment on patella kinematics. Based on a cohort of ten patients examined by dynamic magnetic resonance imaging, their results suggested a more constant patellar tilt in the varus group, which might be associated to a more stable patellar tracking [21]. Consequently, reports discussing potential effects of LOWDFO on patellar tracking are also rare. Swarup et al. showed that the patella congruency angle measured on Merchant views can be improved by LOWDFO in patients with patella instability [14]. Unfortunately, our study lacks pre-operative merchant views giving only limited insight into patella tracking. However, the mean Blackburne-Peel ratios were statistically significantly reduced from pre-operative to postoperative measurements. Considering the relationship of patella instability to patella height (recorded as increased Blackburne-Peel ratios) [22], this could be interpreted as a hint to the potential of LOWDFO to increase patella stability in cases of patella alta in valgus knees. Thus, this interpretation would support the findings of Swarup et al. However, in case of patella instability as well as in case of clinical suspicion of malrotation of the lower limb, a detailed rotational analysis should be considered additionally [23].

Reports on the effect of LOWDFO on leg length are rare. However, the change of leg length is a clinically relevant criterion in patients with a preexisting LLD, when considering a lateral opening wedge or a medial closing wedge distal femoral osteotomy. Interestingly, Madelaine et al. reported that LOWDFO had no effect on leg length [24]. In contrast, our results show that the leg length is significantly increased by LOWDFO. In our cohort, a mean wedge opening height of 9 mm resulted in an increase in leg length of 7.9 mm. A possible explanation of the difference to the results of Madelaine et al. could be a postoperative knee-extension contraction mimicking the increase of leg length. However, our finding is similar to reports on opening wedge high tibial osteotomies [25, 26].

## Conclusion

Lateral opening wedge distal femoral osteotomy without bone grafting using the Tomofix plate is a safe and reliable procedure, which is a promising method particularly in young patients with genu valgum. Positive side effects of the procedure in cases of limb shortage or patella instability are significant leg lengthening and additional patella stability.

## Limitations

Limitations to this study are the retrospective design and the limited cohort size, especially in the subgroup of patients treated with bone grafting, and the subgroup of older patients.
